# De Novo ring chromosome 11 and non-reciprocal translocation of 11p15.3-pter to 21qter in a patient with congenital heart disease

**DOI:** 10.1186/s13039-015-0191-y

**Published:** 2015-11-09

**Authors:** Ying Peng, Ruiyu Ma, Yingjie Zhou, Yan Xia, Juan Wen, Yanghui Zhang, Ruolan Guo, Haoxian Li, Qian Pan, Rui Zhang, Chengyuan Tang, Desheng Liang, Lingqian Wu

**Affiliations:** State Key Laboratory of Medical Genetics, Central South University, 110 Xiangya Rd., Changsha, Hunan 410078 China; The Second Hospital of Hebei Medical University, Shijiazhuang, Hebei P.R. China; Department of Nephrology, The Second Xiangya Hospital, Central South University, Changsha, Hunan P.R. China

**Keywords:** Ring chromosome 11, Non-reciprocal translocation, Congenital heart disease, 11q terminal deletions

## Abstract

**Background:**

Ring chromosome 11[r (11)] is a rare chromosomal abnormality that forms when both arms of chromosome 11 break, and then reunite with each other. Once a ring chromosome forms, the distal ends of both arms of the chromosome are usually lost.

**Case Presentation:**

We reported a 12 years old girl patient with congenital heart disease and distinctive facial features. Cytogenetic and molecular analyses using standard G-banding, fluorescence in situ hybridization and Single nucleotide polymorphism array were performed to identify genetic causes in the patient.

The patient carried r(11)(p15.3q24.1) and 11p15.3-pter non-reciprocal translocation to 21qter, accompanied with 8.9 Mb deletion of 11q24.2q25. A literature review was performed to establish genotype-phenotype correlations of the r (11) and 11q terminal deletion syndrome.

**Conclusions:**

To the best of our knowledge, this is the first case of non-reciprocal translocation with a terminal deletion in r (11). These findings provide important information for genetic counseling for this family, and may improve our understanding of the genotype-phenotype correlation of ring chromosome 11 disorders.

## Background

Ring chromosomes are uncommon cytogenetic abnormalities that have been implicated in all human chromosomes, especially in chromosome 1, 8, 15, X, Y (http://ssmc-tl.com/sSMC.html). Ring chromosomes are formed when both chromosome arms break and then reunite a continuous ring, which is usually accompanied with the loss of some genetic materials [1] Complete ring chromosomes with no apparent deletion of the telomeric ends have aslo been implicated [2]. The clinical manifestations of patients with ring chromosome may be highly variable, which are influenced by many factors, including the loss of genomic materials, the structural instability of rings at mitosis, the rate of secondary aneuploidy cell [1, 3–5].

Ring chromosome 11 [r(11)] is quite rare. Since the first report by Valente M et al. in 1997, only 19 cases of r(11) have been reported [6–16]. Except for two familial r(11) cases [6, 14], the others are sporadic. Difference in clinical features was described among patients carrying r(11), but most cases showed short stature, microcephaly, growth delay, and mental retardation. Most r(11) cases were determined by conventional cytogenetic analysis, molecular analysis was performed in only two reports [6, 16], so precise genotype-phenotype correlations for r (11) remains largely unknown.

We report here a patient carried r(11)(p15.3q24.1) and 11p15.3-pter non-reciprocal translocation to 21qter, accompanied with 8.9 Mb deletion of 11q24.2q25. To the best of our knowledge, this is the first case of non-reciprocal translocation with a terminal deletion in r (11).

## Case presentation

### Patient

The proband, a girl of 12 years old, was the first child of healthy non-consanguineous parents. Her father and mother were 30 and 28 years old at her birth, respectively. After uneventful and full-term pregnancy, the baby was delivered by cesarean section with a birth weight of 2500 g, length 50 cm and head circumference 34.5 cm. Her mother contacted toxic substance mercury during pregnancy. In the neonatal period, the girl was diagnosed with Tetralogy of Fallot, a congenital heart defect. The girl had no pathological jaundice. She could lift her head from a prone position at 3 months, walked at 14 months, and spoke at 15 months. At 2 years old, she underwent surgery to treat heart disease.

**Fig. 1:**
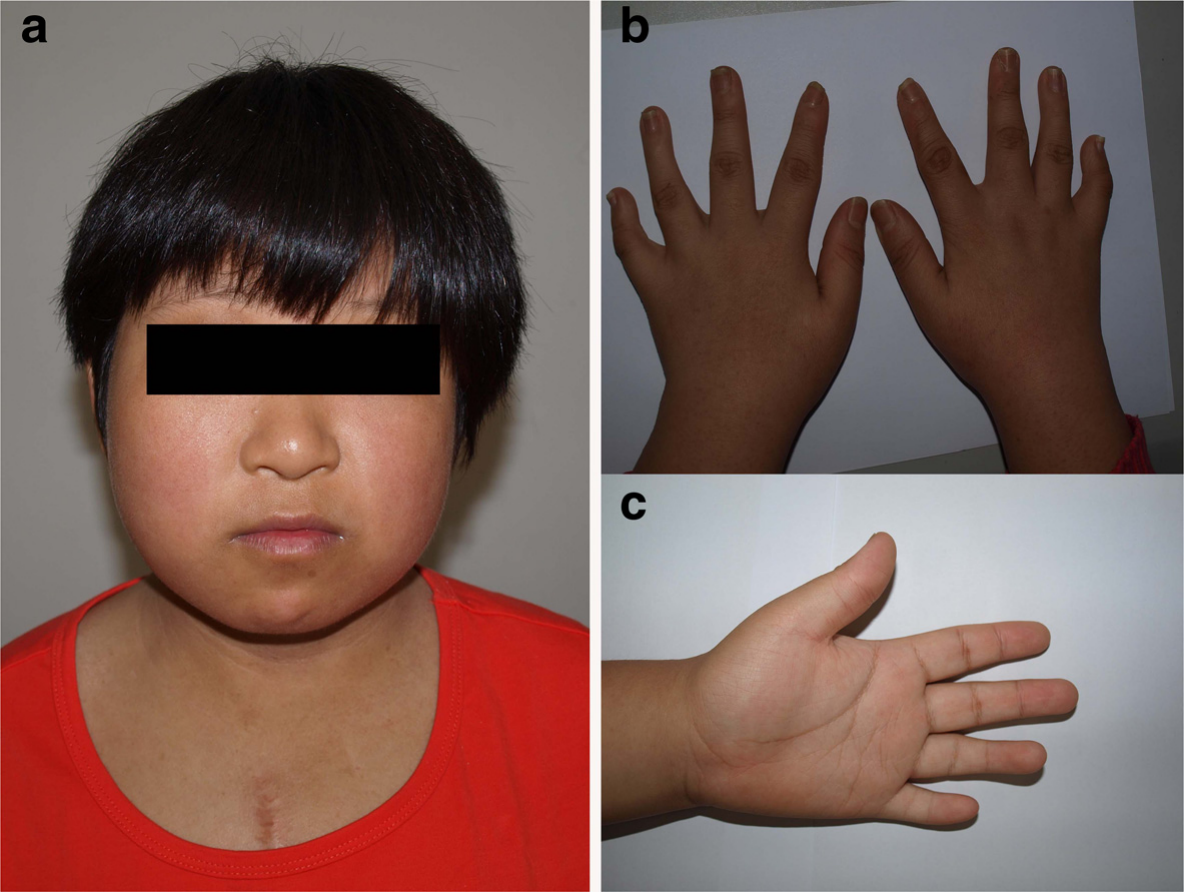
The clinical features of the patient. **a** Frontal view of the patient, showing facial asymmetry, high prominent forehead, small eyes, ocular hypertelorism with droopy eyelids, epicanthal folds, flat nasal bridge, thin upper lip, short neck, and broad thorax. **b**, **c** Small hands, the fifth finger bending and brachydactyly

The patient was fully investigated when she was referred to our clinic genetics at the age of 12 years. Her height was 145 cm (~50th centile), weight 55 kg (~90th centile). She had several dysmorphic features, including facial asymmetry, high prominent forehead, small eyes, ocular hypertelorism with droopy eyelids, epicanthal folds, flat nasal bridge, thin upper lip, short neck, broad thorax, small hands and feet, the fifth finger bent and brachydactyly. There were white spots on her arms and legs. Her language skills were satisfactory, and her motor development was normal. Behavioral problems were noticed, including a short attention span, easy distractibility, and inactive communication with others. The Raven's Standard Progressive Matrices revealed that her intelligence quotient (IQ) was 72. Platelet counts on presentation was 102 × 10^9^/L, which is within normal limits. Platelet morphology with peripheral smear showed no giant platelets or any other abnormality (Fig. 1).

### Cytogenetic and molecular analyses

G-banding (400–550bands) was performed on metaphase chromosomes of cultured peripheral blood lymphocytes obtained from the patient and her parents. FISH analysis was performed according to the manufacturer’s instruction (www.abbottmolecular.com), and the images were captured using a Leica DM500B camera and Leica CW400 software (Applied Imaging). Three bacterial artificial chromosome (BAC) clone probes specific for the region 11p15.4 (RP11-89B9, red), the 11q25 (RP11-7 K8, green), and 21q22.3 (RP11-135B17, orange) were applied. To determine the presence of telomere regions in r(11) and the derivative chromosome 21, FISH using telomere region–specific probes Mix #4 and Mix #11 (Vysis ToTelVysion Multi-Color FISH probe, Abbott, U.S.) was performed. Mix #4 contains specific probes for 4p(chr4: 86938, Spectrum Green), 4q(chr4: 190049608, Spectrum Orange) and 21q(chr21: 47931911, Spectrum Green and Spectrum Orange). Mix #11 contains specific probes for 11p(chr11: 199260, Spectrum Green), 11q(chr11: 134643494, Spectrum Orange) and 18p(chr18: 140409, Spectrum Green and Spectrum Orange).

Genomic DNA was extracted from peripheral blood lymphocytes of the patient using the phenol-chloroform method. Genome-wide copy number analysis was performed using Illumina Human Cyto-SNP12 BeadChip (Illumina, San Diego, CA). The arrays were scanned on a microarray scanner and analyzed using GenomeStudio (cnv Partition Plug-in v3.1.6) software. The operative procedures mentioned above were all performed according to the manufacturer’s recommended protocols (www.illumina.com).

## Results

**Fig. 2:**
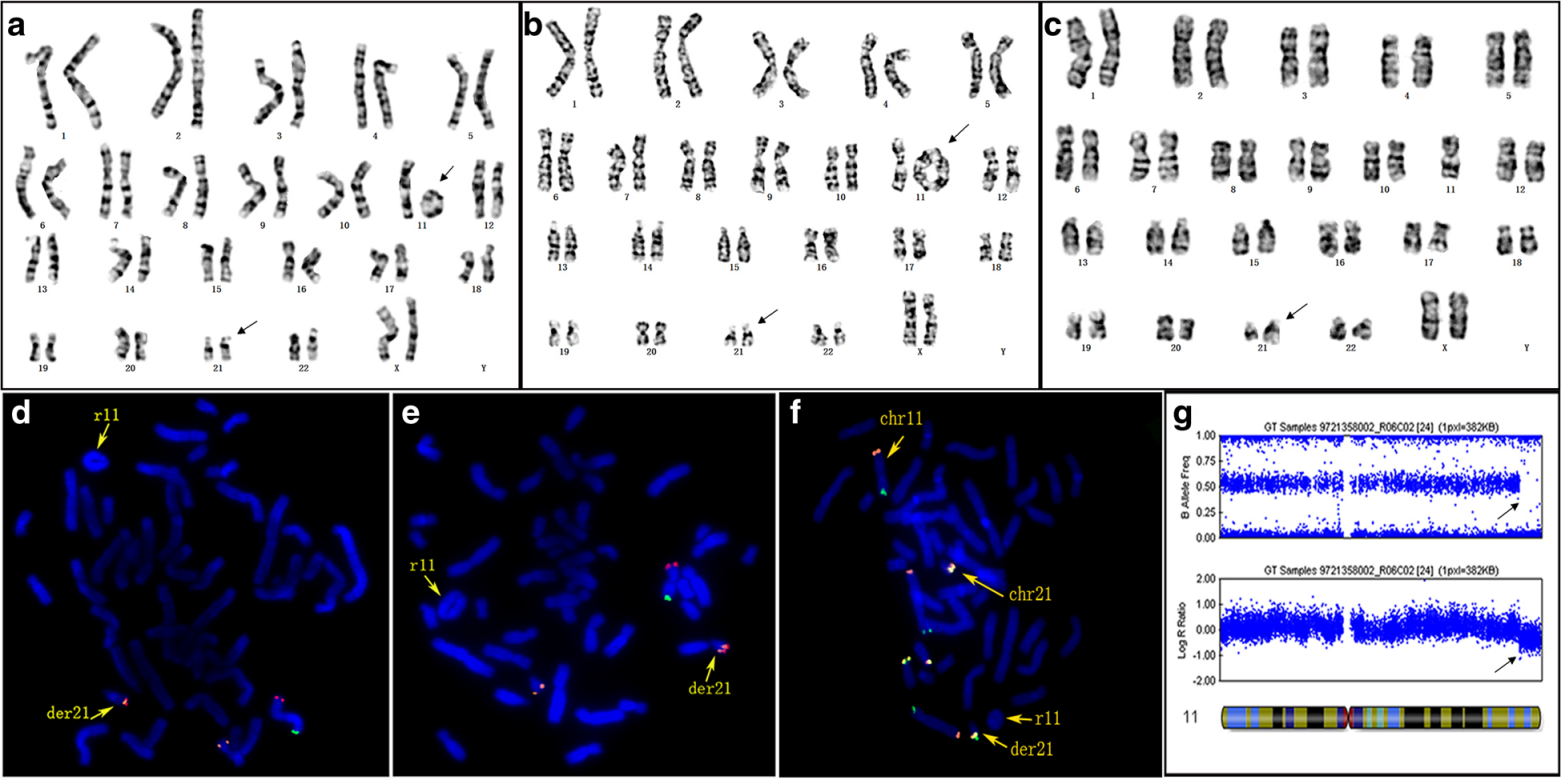
Cytogenetic and molecular analysis results. **a**, **b**, **c** G-banding indicated chimerism of r(11) , r(11; 11) and −11. **d**, **e** FISH using three bacterial artificial chromosome (BAC) clone probes showed a red signal translocation to 21qter and a green signal absent, while the two orange signals still at 21q22.3. **f** FISH using telomeric probes showed two telomeres presented on the derivative chromosome 21q. **g** Copy number variation analysis for individual SNP loci along chromosome 11 showed a 8.9 Mb deletion at 11q24.2q25

G-banding analysis showed the karyotype of the patient was 46, XX, r(11) (p15.3q24.1), der(21) t(11;21) (p15.3;qter) [158] / 45,XX,-11 [16] / 46, XX, r(11; 11) (p15.3q24.1; p15.3q24.1), der(21) t(11;21) (p15.3;qter) [5] (Fig2. a, b, c). The karyotypes of both parents were normal (data not shown). FISH analysis further confirmed the r(11), the absence of 11qter signal and the translocation of 11pter signal to chromosome 21qter (Fig2. d, e). FISH analysis showed there were two telomeres in the derivative chromosome 21qter in the patient (Fig2. f). SNP array analysis detected a 8,915,290 bp deletion in 11q24.2q25 (Fig2. g). Collectively, these finding suggest that the karyotype of the patient is 46,XX,r(11)(p15.3q24.1),der(21)t(11;21)(p15.3;qter)[158] / 45,XX,-11[16] / 46,XX,r(11;11)(p15.3q24.1;p15.3q24.1),der(21)t(11;21)(p15.3;qter)[5]. ish r(11)(p15.3q24.1)(RP11-89B9-,RP11-7 K8-,RP11-135B17-), der(21)t(11;21)(p15.3;qter)( RP11-89B9+, RP11-135B17+). arr 11q24.2q25(126,028,717-134,944,006) × 1dn.

## Discussion

Ring formation and translocation in a same chromosome have been rarely described. So far, only four similar cases have been reported in the literature [17–20], none of which happens in chromosome 11. Here, we reported a special chromosomal rearrangement in a patient carrying r(11) and 11p15.3-pter non-reciprocal translocation to 21qter, accompanied with a terminal deletion at chromosome 11q24.2q25.

Considering this complex rearrangement derived from two different chromosomes 11 and 21,our cellular and molecular analysis suggested that the breaks might happened at two specific time points, either at the time when sister chromatids started to separate during meiosis II or before DNA replication at the interphase of mitosis just after the zygote formation. The different between them was the state of the gamete. In former situation, gamete was abnormal, which contained a r(11) and a der(21). In the latter situation, gamete was normal. But, no matter in which situation, the chimeric karyotype r(11; 11) and −11 could be generated in the subsequent mitosis because of the nondisjunction of duplicate ring. When chromosome breaks take place, the broken chromosomes are highly unstable. To prevent chromosome fusion and exonucleolytic degradation, the broken ends are usually rescued by the addition of telomeric tandem repeat sequence at the breakpoint site or by capturing the telomeric sequences from the same or another chromosome [21, 22]. In addition to telomere healing and capture, Knijnenburg et al. [21] also suggested that ring chromosome formation acted as an alternative mechanism of chromosome rescue in response to chromosome breaks. In a word, chromosome healing has been often involved in chromosome rearrangement, but the specific mechanism remains unclear [23]. In our case, both ring chromosome formation and capturing the telomeric sequences were observed. To prevent exonucleolytic degradation, the breakpoint of chromosome 11p15.3 and 11q24.1 were reunited to form a ring, and the 11p15.3-pter fragment recombined with telomeric sequences of 21qter possibly through nonhomologous end joining (NHEJ) that is the major form of Double-strand breaks (DSBs) repair [24].

The clinical features of our patient included facial dysmorphic features, congenital heart disease and learning difficulties, which also characterized r(11) or 11q terminal deletion syndrome. A summary of the cases of r(11) or 11q terminal deletion syndrome are listed in Table 1. Interestingly, about 70 % patients with r(11) or 11q deletion were female. One potential reason for the phenomenon is that the expression of 11q terminal deletion is somehow influenced by the sex chromosome complement [25].

**Table 1:** A comparison of clinical features between the patient of this study and those previously reported with r(11) and distal del(11q) syndrome

Clinical findings	r(11) (Hansson KB et al. 2012 [6])	Distal del(11q) syndrome (Grossfeld et al. 2004 [31])	Present patient
Number	*n* = 19	*n* = 110	1
Gender	14f/5 m	72f/38 m	f
Age	Ranges from prenatal to adult	Ranges from newborn to adult	12 years
De novo	13	+/94 %	+
Short stature	14	+/68 %	−
Microcephaly	14	N. I.	−
Dysmorphic features	6	+/83 %	+
Short and broad neck	N. I.	+/50 %	+
Mental or development retardation	15 range form mild to severe mental retardation,	+/85 %	Learning difficuties
Congenital heart defect	5	+/56 %	+
Skin	13 with café-au-lait spots, 6 of them with other pigment abnormalities, 5 normal	+/22 % with eczama	Pigment abnormalities
Skeletal abnormality	1	+	+
Kidney abnormality	2 Wilm’s tumor	+/8 %	−
Hematological abnormalities (Paris-Trousseau Syndrome)	N. A.	+/94 %	−
Recurrent infections	N. A.	+/54 %	+
Gastrointestinal	N. A.	+/15 %	−

Clinical findings: +, present; −, absent

*f* female, *m* male, *N. I.* not investigated, *N. A.* not applicable

**Fig. 3:**
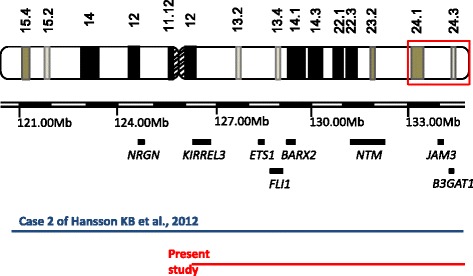
The deletion region of chromosome 11 in the patient and the disease relevant genes within the region

To narrow down potential genetic causes of the clinical features of r(11) or 11q deletion , we compared our data of SNP array analysis with those published. The case two from Hansson et al. [6] was very close to our case. In the case two, no deletion on the short arm of chromosome 11 was noticed, and a considerable deletion region at the terminal part of the long arm of chromosome 11 was detected. The deletion of the case two was 14.1 Mb at 11q23.3q25, which contains a large number of genes, making an appropriate genotype-phenotype correlation difficult. Despite the difference in the size of deletion, both the case two and our case shared some phenotypes. The overlapping region between them encompasses 29 RefSeq genes, including some disease-relevant genes: *JAM3*(OMIM:606871), *ETS1*(OMIM:164720)*, BARX2*(OMIM:604823), *FLI1*(OMIM:193067), *NTM*(OMIM:607938), *KIRREL3*(OMIM:607761) and *B3GAT1*(OMIM:151290) (Fig. 3). Phillips et al. [26] provided evidence that the *JAM3* located at chromosome 11q25 was a strong candidate gene for cardiac phenotype. A crucial role of the *ETS1* gene in heart development has also been reported [27]. Moreover, the haploinsufficiency of the *JAM3* and *ETS1* in patients with cardiac anomalies, and their deletion in our patient with congenital heart disease reinforce the critical role of these two genes in heart development. Meech et al. [28] suggested that the *BARX2*, which encodes an important regulator of muscle growth and repairment, was responsible for facial dysmorphic features, which was supported by our findings. More interestingly, the case two reported by Hansson et al. had severe mental retardation, however our patients showed only some learning difficulties. Four genes within the deletion region of the case two have been associated with nervous system diseases, including *NRGN*(OMIM:602350)*, KIRREL3*, *NTM* and *B3GAT1*. Except for *NRGN*, the other three were also deleted in our cases. These findings suggest that *NRGN* may be a strong candidate gene that is responsible for the mental retardation. *NRGN*, which locates at 11q24.2, encodes a direct target for thyroid hormone in human brain [29]. Moreover, Ohi et al. reported a *NRGN* mutation in 414 Japanese patients with schizophrenia and healthy subjects [30]. They concluded that the genetic risk variant in *NRGN* gene may be associated with the reduction of intellectual ability. Furthermore, Grossfeld et al. concluded that at least 94 % patients with 11q deletion had the Paris-Trousseau Syndrome [31], which is mainly featured by thrombocytopenia, abnormal platelet function and abnormal megakaryocytes. The proto-oncogene *FLI-1*, which maps at chromosome 11q24.3, plays a fundamental role in megakaryocytic differentiation. The haploinsufficiency of *FLI-1* was associated with dysmegakaryocytopoiesis and thrombocytopenia. However, our patient showed no features of Paris-Trousseau Syndrome, although she had the deletion of *FLI-1* gene. We hypothesize that other genes on 11q24 may co-contribute to thrombocytopenia with *FLI-1*. In line with this notion, a recent report by Trkova et al. implicated that a second unknown hit was required to develop thrombocytopenia except for the deletion of *FLI-1* gene [32]. Of course, besides the loss of genetic materials, the configuration of the ring chromosome and epigenetic factors may contribute to the phenotype through influencing gene expression [33–35]. So, these factors also needs to be taken into account in the evaluation of the genetic consequences.

## Conclusions

In summary, this is the first report of r(11) and non-reciprocal translocation of 11p15.3-pter to 21qter, accompanied with a terminal deletion of 11q24.2q25. These findings provided important information for genetic counseling and future clinical treatment for the patient, improve our understanding of the genotype-phenotype connection of r(11), and help further exploring the specific mechanism of chromosome rearrangement.

## Consent

Written informed consent was obtained from the parents of the patient for publication of this Case report and any accompanying images. A copy of the written consent is available for review by the Editor-in-Chief of this journal.

## Abbreviations

BAC: bacterial artificial chromosome
DSBs: double-strand breaks
FISH: fluorescence in situ hybridization
NHEJ: nonhomologous end joining
R (11): ring chromosome 11
SNP array: single nucleotide polymorphism array
